# Correction: Association of SNPs within *TMPRSS6* and *BMP2* genes with iron deficiency status in Saudi Arabia

**DOI:** 10.1371/journal.pone.0306683

**Published:** 2024-07-02

**Authors:** Osama M. Al-Amer, Atif Abdulwahab A. Oyouni, Mohammed Ali Alshehri, Abdulrahman Alasmari, Othman R. Alzahrani, Saad Ali S. Aljohani, Noura Alasmael, Abdulrahman Theyab, Mohammad Algahtani, Hadeel Al Sadoun, Khalaf F. Alsharif, Abdullah Hamadi, Wed A. Abdali, Yousef MohammedRabaa Hawasawi

The 12th author’s name is spelled incorrectly. The correct name is: Abdullah Hamadi.
