# Supplementary material for: Association of SNPs within TMPRSS6 and BMP2 genes with iron deficiency status in Saudi Arabia
Source: PLoS One. 2021 Nov 15;16(11):e0257895. doi: 10.1371/journal.pone.0257895 (PMC8592490; doi:10.1371/journal.pone.0257895)
Supplement: Attachment — Submitted filename: English proofreading letter.docx [file pone.0257895.s001.docx]

**English proofreading letter**

Dear. Chief editor of Journal PLOS ONE,

Please find enclosed research article that we are pleased to submit for publication in Journal of PLOS ONE. Our research paper entitle is “Association of the *BMP2* and *TMPRSS6* genes polymorphisms with iron deficiency status in Saudi Arabia”. This article has been reviewed and heavily edited by a native English speaker Dr. Emily Lenore Goldman Heaphy. Dr. Emily is a Senior Clinical Epidemiologist at Research-Jeddah. Her contact e-mail is :j1513915@kfshrc.edu.sa; emily.lg.heaphy@gmail.com.

Sincerely,

Yousef M. Hawsawi, MSc, Ph.D.

Associate Scientist

King Faisal Specialist Hospital and Research Center- Research Center, KFSH&RC

MBC-J04, P.O. Box 40047- Jeddah, 21499, Saudi Arabia

Tel: +966126677777 Ext. 66217

Fax: +966126634295

E-mail: hyousef@kfshrc.edu.sa / hawsa33@hotmail.com
