# Supplementary material for: Association of SNPs within TMPRSS6 and BMP2 genes with iron deficiency status in Saudi Arabia
Source: PLoS One. 2021 Nov 15;16(11):e0257895. doi: 10.1371/journal.pone.0257895 (PMC8592490; doi:10.1371/journal.pone.0257895)
Supplement: Attachment — Submitted filename: Response to reviewer 17-05-2021.docx [file pone.0257895.s002.docx]

**Letter of Response to Reviewer’s Comments**

**Dear chief editor for PLOS ONE Journal**

**Many thanks for your peer review and comments. With great pleasure, we would like to inform you that, the manuscript has been revised comprehensively and we address all the reviewers' comments. We hope our manuscript satisfied the editor and reviewer and be considered for publication in your decent journal. Our updated response to the reviewers as following:**

1- **Response to Reviewer #1:**

**Sample size is so small for PhD paper**

Many thanks for the important comments and recommendations**. In Saudi Arabia and due to the Arabian culture where females are totally separated from males in the university, it was very difficult to collect samples from the female students. We did our best to collect as much as we can and we finally obtained a total of 108 blood samples from female students. Moreover, for the iron deficient students, we excluded all students diagnosed with eating disorders, pregnant, and those taking nutritional medications or supplements which reduced the number of participants.**

**On which criteria you classified the students as healthy and iron deficient**

Many thanks for the important comments and recommendations**. The criteria for the students as healthy and iron deficient were according to the WHO iron deficiency anemia assessment which was published in 2001. Levels of ferritin < 15 ng/ml and haemoglobin < 12 g/dl were defined as iron deficiency anaemia, whereas levels of ferritin < 15 ng/ml and haemoglobin > 12 g/dl were defined as iron deficiency without anaemia.**

**Have you made any questionnaire for participants? If yes provide it**

**Many thanks for this essential comment. However, because we measured the serum iron level there was no questionnaire for the participants.**

**Provide consent form of participants for provision of blood samples**

**Many thanks for this crucial comment. Consent form of participants for provision of blood samples is attached as requested.**

**Primers used for PCR are previously used or novelty designed**

**Many thanks for this valuable point. The primers were novelty designed.**

**What is m in the first line of discussion?**

**Many thanks for this precise notice. The m was a mistake from the proofreading and it has been removed. The sentence is " was thought to be due to dietary and environmental factors" and it has been highlighted in yellow.**

**Provide the form of ethical committee or institution review board of university.**

**Many thanks for this essential point. Ethical approval (IRB #2018-36) is attached as requested.**

2- **Response to Reviewer #2:**

**The current study tried to assess the effects of TMPRSS6 and BMP2 SNP on the risk of iron deficiency anaemia in adult females from Saudi Arabia. The authors have done a great job in planning and executing this study. Both the study design and the results are is clear laid out. However, the article would require a complete review of the English grammar to make it suitable for publication.**

**Many thanks for this and the manuscript has been sent for proofreading and English grammar was reviewed.**

**1. The title should be reviewed for correctness. For example, “polymorphisms of genes TMPRSS6 and BMP2” may be written as SNPs within TMPRSS6 and BMP2 genes with iron deficiency….**

**Many thanks for this essential comment. The title has been changed according to your valuable advice, and highlighted in yellow.**

**2. Introduction is unnecessarily long. Can be shortened to begin by talking about IDA and exclude general description of iron.**

**This is a valid point and we agree with the reviewer. Therefore, the introduction has been shortened and focused on IDA.**

**3. Why was TMPRSS6 rs4820268 not included in the analysis. This SNP has been widely associated with low iron status in different global populations.**

**Many thanks for this valid point. In our previous publication, we studied the TMPRSS6 rs4820268 SNPs in details.**

**4. Further English editing might help to improve the grammar. For example, the last paragraph of the introduction “….to investigate polymorphism rs1421312 of the TMPRSS6 gene…” may be written as TMPRSS6 rs1421312.**

**Many thanks for this point and the manuscript has been sent for proofreading and English grammar were reviewed. The mentioned sentence has been corrected and highlighted in yellow.**

**5. In section 3.2, it is not clear what this 3-5cc means. It will be helpful to write this use SI units (mL for the blood volume).**

**Many thanks for this point and the 3-5cc was corrected to mL in the manuscript and highlighted in yellow in page 4.**

**6. In the results section, line 1, TMPRSS6 rs1421312 is wrongly written rs141312.**

**Many thanks for this point. The SNP is now corrected as rs1421312 and highlighted in yellow.**

**7. Genotype frequencies of the two SNPs in the study population differed widely from the global and all regional populations including Asian populations in the 1000 Genomes project. I recommend that the authors review their genotyping method to ensure that there were no errors.**

**Many thanks and the genotype frequencies of the two SNPs in the study population was reviewed and there was no error.**

**8. In the section “Genotype distribution of TMPRSS6 rs1421312 and BMP2 rs235756 according to clinical parameters”, the description of the haematology and biochemical parameters using “normal” and “abnormal” should be high and low levels respectively.**

**Many thanks and the description of the haematology and biochemical parameters were changed and highlighted in yellow.**

**9. In page 9, the sentence “Although the exact mechanism is unclear, compelling evidence emphasizes the role of TMPRSS6 polymorphisms in causing IDA”, needs to be reviewed. TMPRSS6 SNPs have been associated with IDA but causality has is not established. Similarly, the grammar in the first sentence of the last paragraph in page 9 needs to be reviewed.**

**Many thanks for this important point. The sentence was reviewed and corrected according to the reviewer advise and highlighted in yellow.**

**10. In the last sentence on page 9, “TMPRSS6 rs141312” the SNP ID is incorrect.**

**Many thanks and the SNP has been corrected as** rs1421312 **and highlighted in yellow.**

**11. The information about the location of BMP2 mentioned in the second sentence of page 10 needs to be reviewed. BMP2 is located in chromosome 20, see Here, but not Chr 2 as mentioned by the authors.**

**Many thanks for this important point, the zero was removed by mistake during the proofreading. Currently, it has been corrected to chromosome 20 and highlighted in yellow.**

**12. The last paragraph in page 10 needs to be reviewed. The sentence “we observed no significant association between increased risk for IDA and SNP rs235756 on BMP2” may be written as ….risk of IDA and BMP2 rs236756….**

**Many thanks for this important point and the sentence has been corrected accordingly.**

**13. The conclusion that the results of this study “can be used as a predictive marker of IDA is Sauda Arabia” is premature. Further research on more genetic markers is needed to verify the clinical usability of this findings. These two SNPs are not sufficient to make this conclusion.**

**This is a valid point and we totally agree with the reviewer and therefore the statement has been removed.**

**Many thanks for your fairly and timely peer review along with valuable comments. We addressed all the highlighted reviewers' comments with a hope that, the revised manuscript will be considered for publication in your decent journal.**

**Looking forward to hearing from you soon.**

**Best regards**

**Yousef Hawsawi**
