# Supplementary material for: Association of SNPs within TMPRSS6 and BMP2 genes with iron deficiency status in Saudi Arabia
Source: PLoS One. 2021 Nov 15;16(11):e0257895. doi: 10.1371/journal.pone.0257895 (PMC8592490; doi:10.1371/journal.pone.0257895)
Supplement: Attachment — Submitted filename: Response second to reviewer 05-09-2021.docx [file pone.0257895.s003.docx]

**Letter of Response to Reviewer’s Comments**

**Dear chief editor for PLOS ONE Journal**

**Many thanks for your distinguish peer review. With great pleasure, we would like to inform you that, the manuscript has been revised for the second round comprehensively and we address all the reviewers' comments. We hope our manuscript satisfied the editor and reviewers and be considered for publication in your decent journal. Our updated response to the reviewers as following:**

1- **Response to Reviewer #1:**

**Reviewer #1: Thanks for addressing all concerns. Refrain dual publication of article. try to meet the criteria of sample size**

Many thanks for the positive comments and recommendations**. We will try to meet the criteria of samples size in our next publication. We highly appreciate your valuable comments.**

2- **Response to Reviewer #2:**

**Reviewer #2: The authors have made significant improvements on the manuscript, and they have responded to the comments raised during the review.**

Many thanks for the important comments and recommendations**. It is highly appreciated.**

**However, there are minor issues that need to be addressed to improve the quality of the article as follows:**

**1. Abstract**

**a. 3 – 5 cc in the methods section could be changes to 3 – 5mL.**

Many thanks for this important point. The “3-5 cc” in the methods section has been changed to 3-5 mL.

**2. Introduction section:**

**a. The sentence “….developing and low-income countries” could be change to “****low- and middle-income countries”.**

Many thanks for this important comments, the sentence has been changed to low- and middle-income countries.

**b. In line 3 of the third paragraph, the T in TMPRSS6 is not** **italised..**

**Many thanks for this precise comment and the T has been changed into italised.**

**3. Results**

**a. In the last sentence of the last paragraph, it is not clear what the authors meant by … “associated with recessive genotypes”. The phrase may be removed.**

**Many thanks for this essential comment. The phrase has been removed and the sentence changed to “associated with increased risk for IDA in the female students in the study”.**

**4. Discussion section**

**a. The beginning of paragraph 3: “****McLaren and his colleagues studied SNP in gene TMPRSS6 in four different ethnic groups…” should be revised. Also, the authors may consider removing the pronoun “his” when referring to other authors. Such sentences may be written as e.g.** **McLaren and colleagues studies.**

**Many thanks for this important comment and the “McLaren and his colleagues studied SNP in gene TMPRSS6 in four different ethnic groups…” has been be revised and changed. The sentence McLaren and his colleagues has been corrected to McLaren and colleagues studies.**

**b. In paragraph 4, line 4, the “H” in HFE should be italised.**

**Many thanks for this precise comment and the H has been changed into italised and highlighted in yellow**

**c.** **Millet and his colleagues, see above comments**

**Many thanks for this important comment. The** **Millet and his colleagues has been changed to Millet and colleagues and highlighted in yellow.**

**d. In line 6, it is not necessary to include the P value 0.002 in discussion here and in other places.**

**Many thanks for this important comment, the P values were removed.**

**e. In paragraph 5, lines 4 – 5, the sentence “… direct association between TMPRSS6 and increase risk of IDA” should be revised. The association reported by these authors is between specific TMPRSS6 SNPs and IDA but not TMPRSS6 only..**

**Many thanks for this important comment, the sentence has been corrected and the SNPS were included.**

**5. Additional general comments**

**1. Table 5 need reformatting. In the Odd ration column, there should be a space between the OR and the confidence interval e.g 3.74(0.19-73.05) should be written as 3.74 (0.19-73.05).**

**The table has been space was added and the column was updated.**

**2. There should be a footnote to explain what “ref.” means**

**The footnote was added.**

**3. Table 6 should be on one page. And see the above comments regarding footnotes and brakets.**

The footnote was added and the space also was added.

**Many thanks for the fairly and timely peer review along with valuable comments. We addressed all the highlighted reviewers' comments with a hope that, the revised manuscript will be considered for publication in your decent journal.**

**Looking forward to hearing from you soon.**

**Best regards**

**Yousef Hawsawi**

Additional general comments

1. Table 5 need reformatting. In the Odd ration column, there should be a space between the OR and the confidence interval e.g 3.74(0.19-73.05) should be written as 3.74 (0.19-73.05).

2. There should be a footnote to explain what “ref.” means

3. Table 6 should be on one page. And see the above comments regarding footnotes and brakets.

7. PLOS authors have the option to publish the peer review history of their article (what does this mean?). If published, this will include your full peer review and any attached files.

If you choose “no”, your identity will remain anonymous but your review may still be made public.

Do you want your identity to be public for this peer review? For information about this choice, including consent withdrawal, please see our Privacy Policy.

Reviewer #1: No

Reviewer #2: Yes: Momodou W. Jallow, PhD.
